# Identification of ClpB, a molecular chaperone involved in the stress tolerance and virulence of *Streptococcus agalactiae*

**DOI:** 10.1186/s13567-024-01318-6

**Published:** 2024-05-15

**Authors:** Lan Yang, Zhihao Wu, Tian-Yu Ma, Hui Zeng, Ming Chen, Yong-An Zhang, Yang Zhou

**Affiliations:** 1 National Key Laboratory of Agricultural Microbiology; Hubei Hongshan Laboratory; Engineering Research Center of Green Development for Conventional Aquatic Biological Industry in the Yangtze River Economic Belt, Ministry of Education; Shenzhen Institute of Nutrition and Health, College of Fisheries, Huazhong Agricultural University, Wuhan, 430000 China; 2 Guangdong Laboratory for Lingnan Modern Agriculture, Guangzhou, 510000 China; 3 Shenzhen Branch, Guangdong Laboratory for Lingnan Modern Agriculture, Genome Analysis Laboratory of the Ministry of Agriculture,, Agricultural Genomics Institute at Shenzhen, Chinese Academy of Agricultural Sciences, Shenzhen, 518000 China

**Keywords:** ClpB, *Streptococcus agalactiae*, stress tolerance, virulence, *Oreochromis niloticus*

## Abstract

Bacterial ClpB is an ATP-dependent disaggregate that belongs to the Hsp100/Clp family and facilitates bacterial survival under hostile environmental conditions. *Streptococcus agalactiae*, which is regarded as the major bacterial pathogen of farmed Nile tilapia (*Oreochromis niloticus*), is known to cause high mortality and large economic losses. Here, we report a ClpB homologue of *S. agalactiae* and explore its functionality. *S. agalactiae* with a *clpB* deletion mutant (∆*clpB*) exhibited defective tolerance against heat and acidic stress, without affecting growth or morphology under optimal conditions. Moreover, the Δ*clpB* mutant exhibited reduced intracellular survival in RAW264.7 cells, diminished adherence to the brain cells of tilapia, increased sensitivity to leukocytes from the head kidney of tilapia and whole blood killing, and reduced mortality and bacterial loads in a tilapia infection assay. Furthermore, the reduced virulence of the ∆*clpB* mutant was investigated by transcriptome analysis, which revealed that deletion of *clpB* altered the expression levels of multiple genes that contribute to the stress response as well as certain metabolic pathways. Collectively, our findings demonstrated that ClpB, a molecular chaperone, plays critical roles in heat and acid stress resistance and virulence in *S. agalactiae*. This finding provides an enhanced understanding of the functionality of this ClpB homologue in gram-positive bacteria and the survival strategy of *S. agalactiae* against immune clearance during infection.

**Supplementary Information:**

The online version contains supplementary material available at 10.1186/s13567-024-01318-6.

## Introduction

*Streptococcus agalactiae*, also referred to as Group B *Streptococcus* (GBS), is a gram-positive bacterium with a broad spectrum of hosts. GBS is the leading infectious cause of early-onset neonatal sepsis and is being increasingly recognized as a cause of stillbirth and preterm birth in humans [1–3]. However, GBS not only affects humans but also has a severe impact on farmed fish, including Nile tilapia (*Oreochromis niloticus*), giant grouper (*Epinephelus lanceolatus*), ya-fish (*Schizothorax prenanti*), Asian seabass (*Lates calcarifer*), Amazon catfish (*Pseudoplatystoma* sp.), golden pompano (*Trachinotus ovatus*), and wild mullet (*Liza klunzingeri*) [4–6]. GBS is considered the main bacterial pathogen of cultured bacteria tilapia, with morbidity and mortality exceeding 80% in outbreaks [7, 8]. In tilapia, the symptoms of streptococcosis include erratic swimming, exophthalmos, skin lesions, and meningoencephalitis [9, 10].

To successfully infect a range of hosts, GBS has evolved to rapidly adapt to changing environmental conditions, including pH, temperature, and osmolality; it is possible that these conditions trigger a stress response in bacteria [11]. To combat invasive pathogens during streptococcal infection, chemokines are known to attract macrophages and neutrophils to the site of infection. In macrophages, bacteria are engulfed by phagosomes, in which lysosomes produce oxidative and acidic species to kill the bacteria [12]. However, according to previous studies, GBS can remain unharmed in macrophages for several hours [13], suggesting that this bacterium has the ability to withstand intracellular conditions in macrophages. Macrophages have been identified as potential “Trojan horses” that help pathogens breach the blood‒brain barrier (BBB) and enter the central nervous system, ultimately leading to meningitis [14, 15].

Heat shock proteins (Hsps) are proteins that play essential roles in stress responses and function as molecular chaperones to stabilize proteins and aid protein refolding under stressful circumstances [16]. ClpB (Hsp100), one of the major bacterial molecular chaperones, is expressed in bacteria, protozoa, fungi, and plants but not in animals or humans [11]. ClpB has the ability to disaggregate stress-denatured proteins along with the DnaK system, thus protecting bacteria from a range of stressors, including heat, acidity, and oxidation [17, 18]. Over the past few decades, ClpB has been investigated in both gram-negative and gram-positive bacteria, revealing its role in stress responses and virulence [19]. In the case of *Francisella novicida*, a bacterium commonly used as a laboratory model for tularemia, deletion of the *clpB* gene not only increased susceptibility to high temperatures but also attenuated intracellular replication in J774A.1 cells [20]. However, the role of ClpB in the stress response and virulence of *Streptococcus* species has yet to be investigated.

In this study, the SAHN016_02930 protein was annotated as an ATP-dependent ClpB protease in the GBS strain HN016. To investigate the biological functionality of ClpB during GBS infection, we generated a *clpB* gene deletion strain and then assessed the sensitivities of the mutant strain to multiple stress conditions. In addition, we evaluated the contribution of ClpB to the virulence of GBS by performing a range of ex vivo and in vivo experiments. Prokaryotic transcriptome sequencing was also performed to analyse the possible regulatory network regulated by *clpB* during macrophage survival.

## Materials and methods

### Bacterial strains, plasmids, cell lines, and growth conditions

The bacterial strains, plasmids, and cell lines used in this study are described in Additional file 3. The GBS strain of HN016 was isolated in 2010 from diseased tilapia in Guangdong Province, China [21]. Strains of GBS were cultured in Todd–Hewitt broth (THB) (Hopebio, China) supplemented with 1.5% (wt/vol) agar at 28 °C. *Escherichia coli* strain DH5α (Trans, Beijing, China) was grown in Luria-Bertani broth (LB) (Hopebio, China) supplemented with 1.5% (wt/vol) agar at 37 °C. The thermosensitive pSET4 suicide vector was used for gene mutation [22]. The antibiotic spectinomycin (Spc) (Biofroxx, Germany) was added to the medium at 100 μg/mL for GBS or 50 μg/mL for *E. coli*, as needed. Murine leukaemia cells from monocyte macrophages (RAW264.7) [26] were cultured in Dulbecco’s modified Eagle medium (DMEM; Gibco, USA) supplemented with 10% foetal bovine serum (FBS; Sigma‒Aldrich, USA) at 37 °C with 5% CO_2_. Tilapia brain (TiB) cells were cultured in DMEM supplemented with 10% FBS at 28 °C. TiB cells, a fibroblast line derived from the brains of tilapia, were a gift from Foshan University [23].

### Bioinformatics analysis

The primary protein sequences of ClpB from GBS (GenBank accession no. AKT95669.1), *E. coli* (GenBank accession no. CAD6006397.1), *Enterococcus faecalis* (GenBank accession no. CWW57247.1) and *Francisella tularensis* (GenBank accession no. AFB79746.1) were downloaded and retrieved from the National Centre for Biotechnology Information (NCBI) databases. The amino acid sequences of the ClpB proteins were compared via Clustal Omega software online. ESPript 3.0 was used to perform various tasks in predicting the secondary structure and sequence homology [24]. Tertiary structures were predicted by SWISS-MODEL and confirmed via PROCHECK [25]. Additional file 4 provides details about the websites, bioinformatics analysis software, and databases used in this study.

### ATPase activity of ClpB

The recombinant ClpB was diluted to different concentrations (0.1 μmol/L-5 μmol/L) and added to working buffer (100 mmol/L Tris-HCl [pH 8.0], 10 mmol/L MgCl_2_, 5 mmol/L ATP, 1 mmol/L EDTA, and 1 mmol/L dithiothreitol) in the absence/presence of α-casein (0.25 mg/mL) for 15 min at 37 °C. Then, ClpB-ATPase activity was measured by estimating the concentration of inorganic phosphate produced from ATP by using the malachite green colorimetric assay [26].

### Construction of the mutant strain

To construct the GBS *clpB* deletion mutant (∆*clpB*), the upstream and downstream fragments of *clpB* were amplified with the primers *clpB*-A/B and *clpB*-C/D, respectively, using gDNA from GBS HN016 as the template. To amplify the *clpB*-OP fusion fragment, overlapping extension PCR was performed using the upstream and downstream fragments as templates with the primers *clpB*-A/*clpB*-D. The restriction enzymes SmaI and SalI were used to digest the fusion fragment and pSET4s, and T4 DNA ligase was subsequently used to construct the *clpB* deletion vector pSET4s-*clpB*. The possible vectors were transformed into *E. coli* DH5α for propagation and verified by colony PCR. Prior to electroporation into GBS HN016 competent cells, pSET4s-*clpB* were sequenced, and the cells were selected with Spc. To confirm whether the deletion affects the expression of upstream and downstream genes, qRT‒PCR targeting the upstream and downstream genes was performed using cDNA from the strain with the identified ∆*clpB* gene as a template. The primers used in this study are described in Additional file 5.

### Growth rate analysis

Bacteria were grown to the mid-log phase (OD_600_ = 0.6) and diluted to OD_600_ = 0.1. Three technical duplicates of 200 μL of each culture were added to a 96-well microplate for OD_600_ measurements. The microplate was incubated at 28 °C, and the OD_600_ was measured automatically by a microplate reader (BioTek Epoch 2, USA) every 30 min with shaking for 10 s before measurement. The average of the replicates is represented as the OD_600_ against time.

### Gram staining

Bacteria were collected and washed twice with phosphate-buffered saline (PBS; Solarbio, China). Twenty microlitre samples were fixed on glass slides (25 mm × 75 mm and 1 mm in thickness; Citotest, China). A Gram Staining Kit (Solarbio, China) was used according to the manufacturer’s instructions. Light microscopy was used to observe the stained samples. In the images, 50 chains of each strain were selected randomly and measured.

### Transmission electron microscopy (TEM)

TEM was performed as previously described [27]. Bacteria were collected, washed twice with PBS, and fixed in 2.5% glutaraldehyde overnight at 4 °C. Once the samples were embedded and sliced, they were observed by TEM (H-7650, HITACHI, Japan) and then randomly selected for capsule measurement.

### Sensitivity of GBS strains to stress

To explore the function of ClpB in stressful environments, the strains were subjected to various stresses using a method described previously [28]. Bacterial cultures were centrifuged, washed, resuspended in PBS, and then incubated at 45 °C for 15 or 30 min. For the acidic assay, the bacteria were collected, washed, and suspended in PBS at different pH values (3, 5, and 7) for 1 h at 28 ℃. The existing cells were spread on THB plates at the appropriate dilutions and incubated at 28 ℃. The proportion of survivors was used to calculate the survival rate.

The response of the GBS strains to oxidative and osmotic stress was assessed as previously described [29]. The cultures at the mid-log phase were diluted to OD_600_ = 0.1 in THB supplemented with 1 mM H_2_O_2_ or 500 mM NaCl. The OD_600_ measurements were processed as described in the subsection “Construction of the mutant strain”.

### Adhesion and invasion assays

TiB cells were used to conduct adhesion and invasion assays as previously reported [30]. In the adherence assay, bacteria suspended in DMEM were added to TiB cells at a multiplicity of infection (MOI) of 10 and then incubated at 28 °C for 2 h. Following three PBS washes, sterile distilled water was used to lyse the infected cells. Adherent bacteria were diluted appropriately and plated on THB agar plates. The invasion assay was performed similarly to the adhesion assay. Before the final lysis, the infected cells were cultured for an additional 2 h in DMEM containing penicillin and streptomycin to kill the extracellular bacteria. The serially diluted lysates were plated and incubated at 28 °C overnight for colony counting.

### Intracellular viability in macrophages

As previously described, the phagocytosis assay was conducted in RAW 264.7 cells [31]. GBS strains were resuspended in DMEM, added to RAW 264.7 cells at an MOI of 1/10/100, and incubated at 37 °C for 1 h. Then, extracellular bacteria were eliminated by incubation in DMEM containing penicillin and streptomycin for 1 h. The cells containing phagocytic bacteria were cultured for another 4, 8, 12, or 24 h, followed by washing, disruption, and viable cell counting.

### Whole-blood bactericidal assays

As previously described, the bactericidal activity was tested in tilapia whole blood [32]. Bacteria were washed and resuspended in PBS containing 1% FBS, and the strains (100 μL) were mixed with 900 μL of fresh whole blood from tilapia that had been heparinized. The mixture was then incubated for 90–180 min at 28 °C with several rotations. The mixtures were plated and incubated at 28 °C to count the viable bacteria. The fold change of bacteria in the blood was expressed as the bacterial load after incubation compared to that in the initial inoculum.

### Leukocyte bactericidal assays in tilapia

The head kidney of tilapia was dissociated into cell suspensions in DMEM and then dispersed through nylon mesh (40 μm; BD Biosciences, USA). Using previously reported methods [8, 33, 34], head kidney leukocytes (HKLs) were separated using 51/34% discontinuous Percoll (GE Healthcare, China) density gradients. Then, the GBS strains in the mid-log phase were centrifuged and resuspended in DMEM. At an MOI of 10, HKLs were added to the bacteria, which were subsequently cultured at 28 ℃ with 5% CO_2_ for 2/3 h. After the addition of Triton X-100 (0.02%) to lyse the cells, the cultures were plated after serial dilution and incubated overnight at 28 ℃.

### Pathogenicity studies in tilapia infection

Bacteria were collected, centrifuged, and resuspended in PBS. After anaesthesia, the tilapia were then intraperitoneally (i.p.) injected with 2 × 10^8^ CFU/fish. Sixty fish were randomly divided into 2 groups of 30 fish each. The infected fish were monitored and fed twice a day for 14 days in individual tanks where the water temperature was maintained at 34 ℃. This experiment was conducted twice.

To better evaluate the pathogenicity of Δ*clpB*, we determined the number of viable bacteria in the organs. A total of 20 fish were divided into 2 groups at random and i.p*.* injected with strains at a dose of 5 × 10^5^ CFU/fish. At 12 h postinfection, brain, spleen, and blood samples were collected, weighed, and homogenized in PBS. The samples were then serially diluted and plated for colony counting.

### Transcriptomic analysis

Bacteria were resuspended and incubated with RAW264.7 cells at 37 °C for 1 h. After removing the extracellular bacteria, the samples were centrifuged. RNA extraction, library preparation, and Illumina HiSeq sequencing were performed on an Illumina NovaSeq 6000 platform at the Shanghai Biozeron Bioinformatics Center (China). RPKMs (reads per kilobase per million reads) were used to determine the gene expression level of each transcript.

### Quantitative real-time polymerase chain reaction (qRT‒PCR) analysis

qRT‒PCR was performed as previously reported [34]. Total bacterial RNA was extracted and reverse transcribed into cDNA. A CFX Connect^™^ Real-Time System (Bio-Rad, USA) was used for qRT‒PCR analysis, and each sample was analysed in triplicate. The 16S rRNA housekeeping gene was used as the internal control gene when determining the gene expression level via the 2^−ΔΔCt^ method [35]. The qRT‒PCR primers used are listed in Additional file 5.

### Statistical analysis

The experiments were repeated three times. The measured experimental data are expressed as the mean ± standard deviation. Using the software GraphPad Prism 8.0, a two-tailed unpaired Student’s *t* test was used to evaluate the *P* value. Statistical significance was defined as *P* < 0.05.

## Results

### Identification of a ClpB homologue in GBS

In the genome of the GBS strain HN016, we annotated the SAHN016_02930 protein as a ClpB protease. ClpB_GBS_ consists of 753 amino acids, compared to the 857 amino acids of *E. coli* ClpB (ClpB_Ec_). Multiple sequence alignment of amino acids showed that ClpB_GBS_ is similar to homologues of *E. coli* (40.2% identity), *E. faecalis* (39.7% identity), and *F. tularensis* (40.2% identity). To evaluate the distribution of ClpB among GBS strains, we performed a BLASTP search of the NCBI database and found that ClpB was conserved across various GBS strains, with protein homology ranging from 99.60 to 100% (Additional file 6).

**Figure 1:**
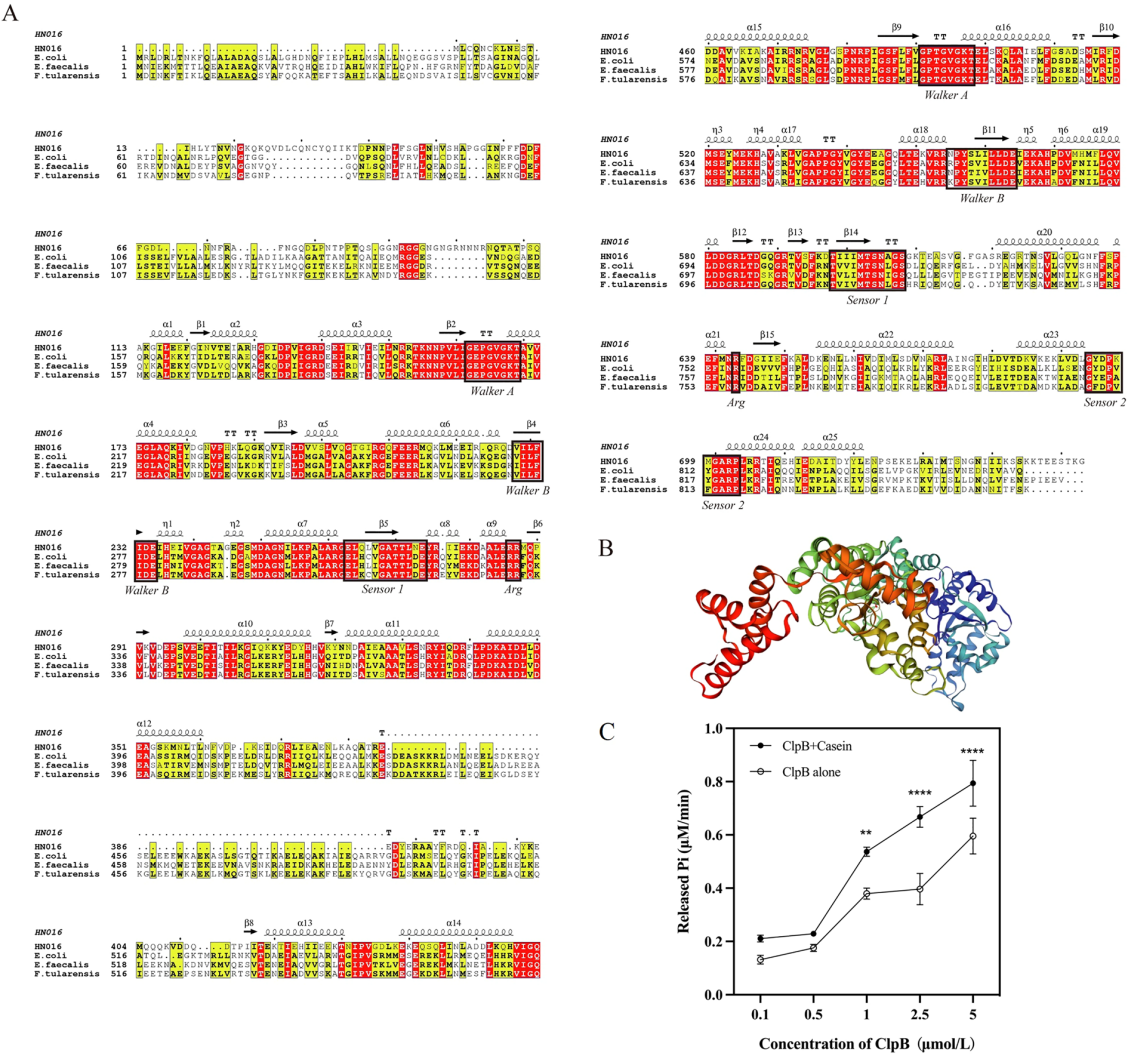
**Bioinformatics analysis of the ClpB protein.** **A** Multiple sequence alignments of ClpB in GBS HN016 and other ClpB isoforms (*E. coli*/*E. faecalis*/*F. tularensis*). The conserved Walker A/B motifs, sensor 1/2 motifs and arginine residues are boxed. **B** The tertiary structure of ClpB predicted by SWISS-MODEL software. ND (blue), NBD-1 (sky blue), MD (green), NBD-2 (orange), and CD (red). **C** ATPase activity of rClpB_GBS_.

Sequence alignment of ClpB_GBS_ revealed that this protein has a multidomain organization similar to that of ClpB_Ec_, including one N-terminal domain (ND), two nucleotide-binding domains (NBD-1/-2), a middle coiled-coil domain (MD), and a C-terminal domain (CD) (Figure 1A). The two NBDs are the most highly conserved, and the main differences are in the ND and the CD. The prediction model of ClpB_GBS_ showed that its reference template protein was 8a8u.1.D (SWISS-MODEL template library), and the template coverage was 82% (Figure 1B). The reasonability of the prediction model was confirmed by PROCHECK (Additional file 1).

### ATPase activity of ClpB

Amino acid sequence analysis revealed the presence of two conserved NBDs in ClpB_GBS_ that are involved in ATP binding. We analysed the ATPase activity of rClpB_GBS_ in the presence and absence of α-casein, which revealed that rClpB_GBS_ exhibited ATPase activity within a concentration range of 0.1–5 μmol/L. However, the ATPase activity of rClpB_GBS_ increased in the presence of α-casein (Figure 1C).

### ClpB was not required for growth or cellular morphology under optimal conditions

**Figure 2:**
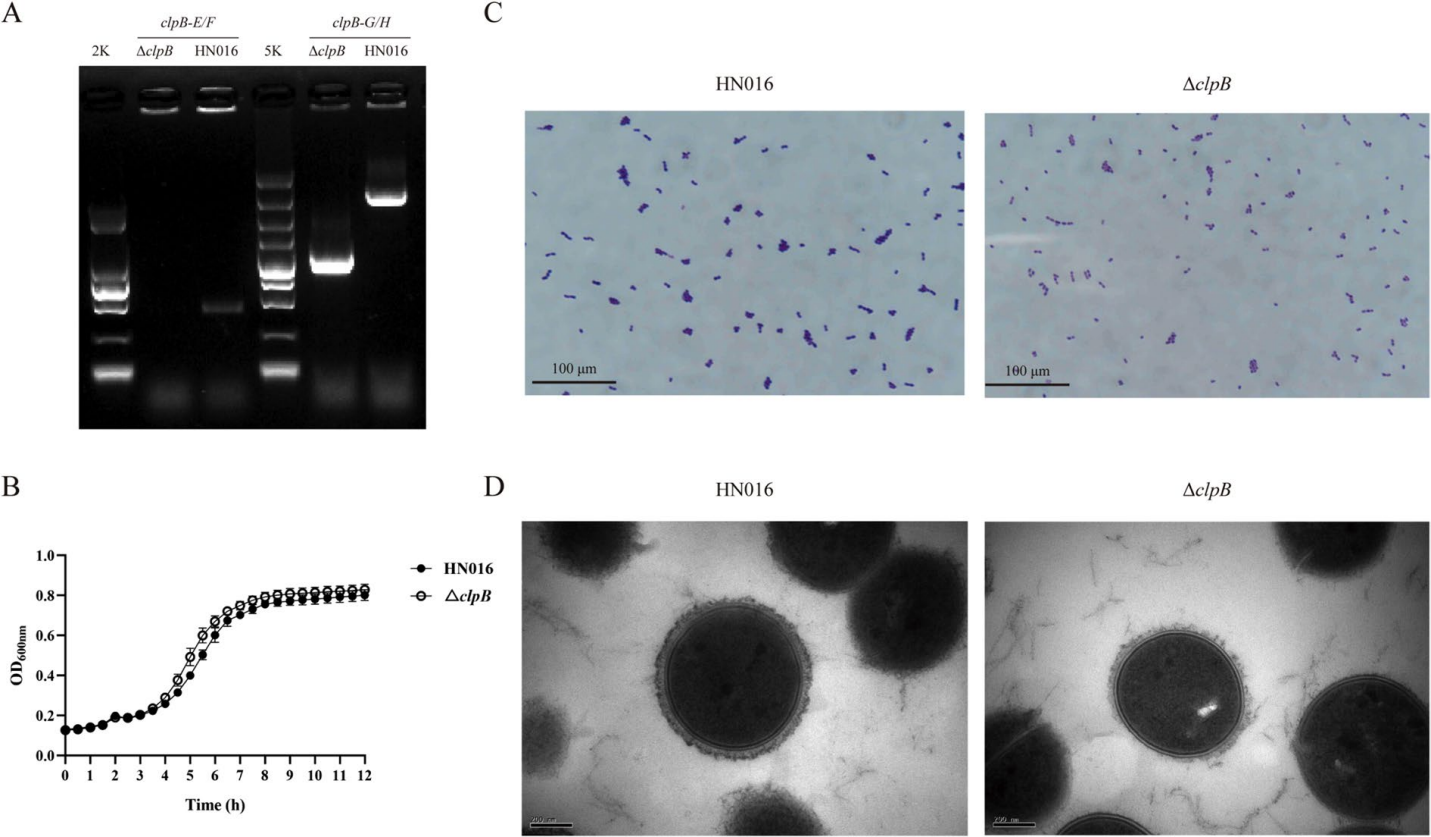
**Construction and biological characterization of ΔclpB.** **A** PCR confirmation of *c*Δ*clpB* using the primers *clpB*-E/F and *clpB*-G/H. **B** Growth curves of GBS strains cultured in THB at 28 ℃. **C** Gram staining and microscopic observation of the WT and Δ*clpB strains*. **D** Transmission electron microscopy of the WT and Δ*clpB strains*.

To investigate the role of ClpB in GBS, we constructed a *clpB* deletion mutant of GBS HN016 by homologous recombination, which was confirmed by PCR. We used an external test primer (*clpB*-G/H) to amplify a 1034 bp fragment from Δ*clpB,* a 3.1 kb fragment from the wild-type (WT) strain, and an inner test primer (*clpB*-E/F) to amplify a 500 bp fragment from the WT; the inner test primer did not amplify a fragment from Δ*clpB* (Figure 2A). The expression levels of the *clpB* gene were also verified by qRT‒PCR in the WT and Δ*clpB* strains. The qRT‒PCR results showed that the *clpB* gene was expressed in the WT but was not expressed in the deletion strain, further confirming that the deletion strain had been successfully constructed (Additional file 2).

To investigate the regulatory impact of *clpB* on the growth of GBS, we generated growth curves by measuring the OD_600_. The growth rates of the Δ*clpB* and WT strains were not significantly different when they were cultured in THB at 28 ℃ (Figure 2B), indicating that, in a suitable environment, the deletion of the *clpB* gene did not impair the growth of GBS.

To investigate morphological variations between the two strains, we carried out Gram staining and TEM to measure chain length and capsule thickness. Bacteria were grown to the mid-log phase, stained with crystal violet, and imaged. On average, Δ*clpB* had a similar chain length, with an average length of 8.9 μm per chain, compared to the average length of 9.3 μm per chain in the WT (*P* > 0.05) (Figure 2C). TEM further revealed that the mean capsule thicknesses for the Δ*clpB* and WT strains were 49.2 and 41.2 nm, respectively; these values were not significantly different (*P* > 0.05) (Figure 2D). Thus, our findings indicate that the deletion of *clpB* had no influence on the morphology of GBS under optimal conditions.

### The ClpB protein contributes to heat and acid stress resistance but not to oxidative or osmotic stress

**Figure 3:**
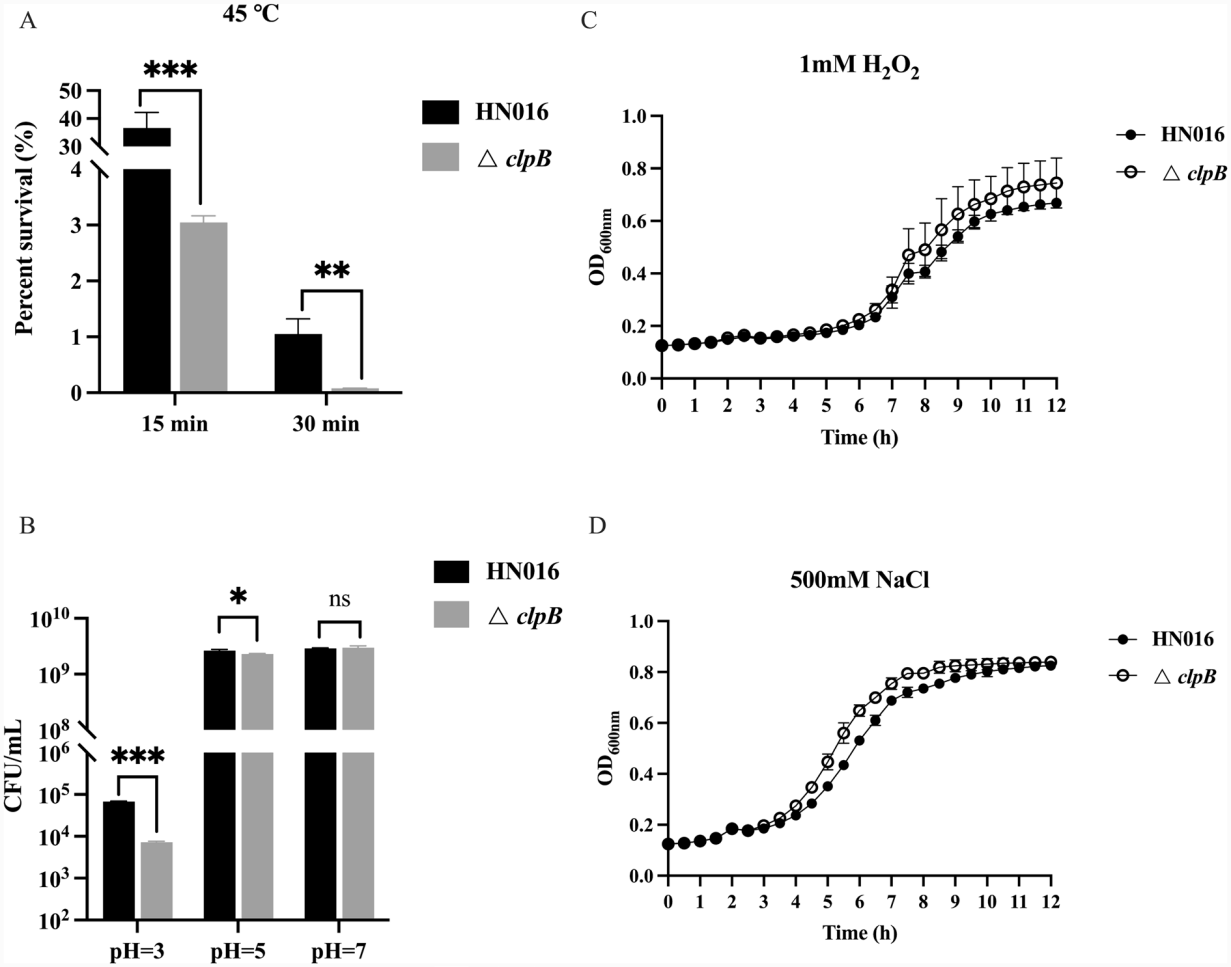
**Bacterial viability and growth curves of the WT and ΔclpB strains.** Surviving colonies were quantified under thermo (**A**) and acidic (**B**) conditions; OD_600_ values were recorded under oxidative (**C**) and osmotic (**D**) conditions. ns, not significant; *P < 0.05, **P < 0.01, ***P < 0.001.

GBS can infect a diverse range of hosts, which implies that this pathogen can adapt to various environments. In this study, the GBS strains were exposed to a variety of stress conditions, including heat, acidic pH, and oxidative and osmotic stress, during in vivo infection experiments. In the heat stress study, compared with that of the WT, the survival rate of the Δ*clpB* mutant significantly decreased in response to heat stress (45 °C), suggesting that ClpB contributes to the thermotolerance of GBS (Figure 3A). Under acidic conditions, the survival of the two strains showed remarkable variations when the pH was 3 or 5. Compared to that of the WT strain (6.8 × 10^4^ CFU/mL), the CFU of the Δ*clpB* strain decreased significantly at pH 3 (7.2 × 10^3^ CFU/mL). However, the survival rates (%) were comparable between the strains at pH 7 (Figure 3B).

Given that ClpB contributes to tolerance under heat and acidic conditions, we next investigated the potential involvement of ClpB in oxidative or osmotic stress (Figures 3C and D). The growth curves for Δ*clpB* did not differ significantly from those for the WT, indicating that ClpB had no effect on the response of GBS to oxidative or osmotic stress.

### ClpB plays a key role in virulence

**Figure 4:**
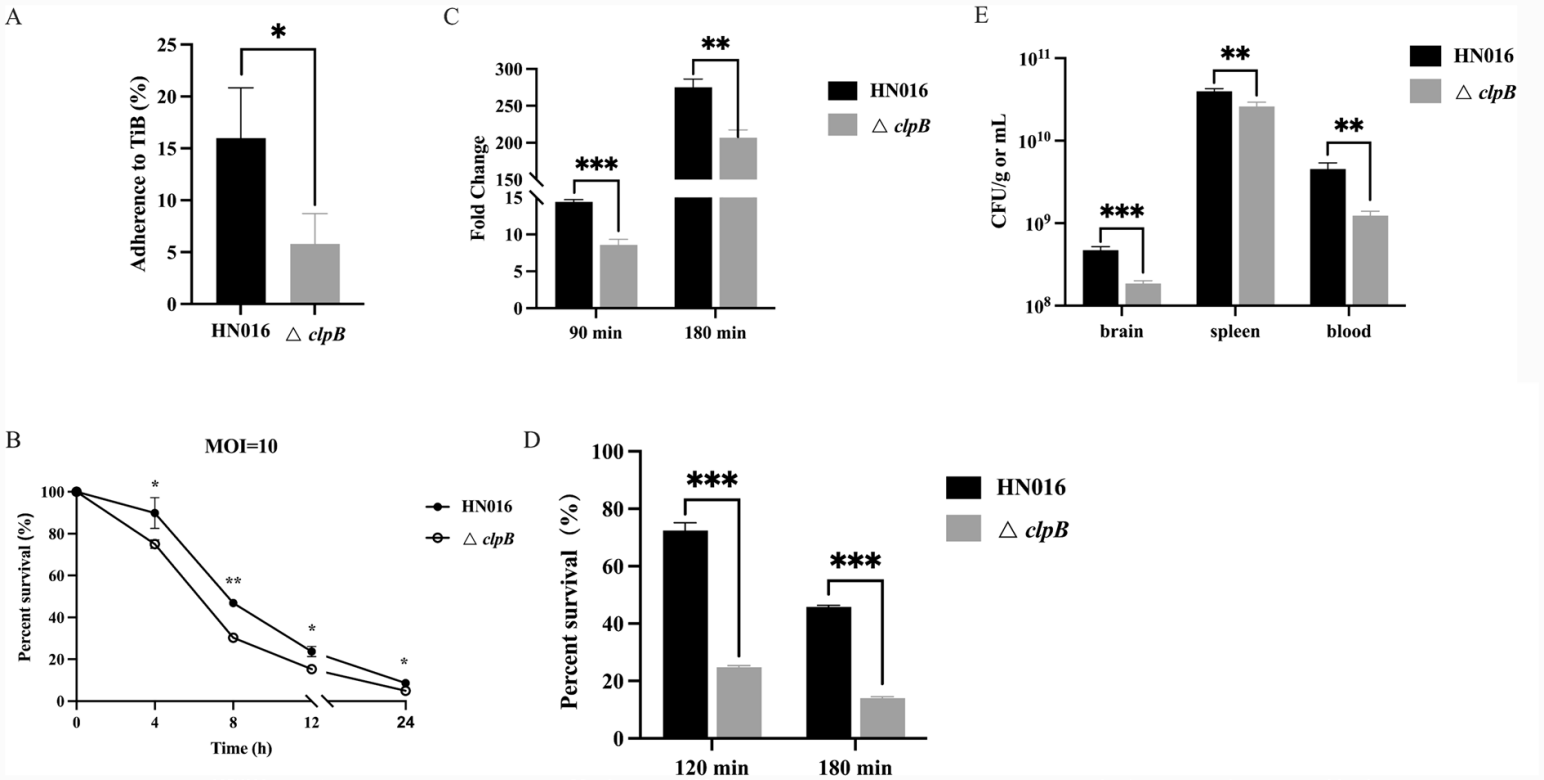
**Effects of clpB gene deficiency on the pathogenicity of GBS** **A** Adherence testing of WT and Δ*clpB* strains to TiB cells. **B** Phagocytosis assays were performed on RAW 264.7 cells. **C** Multiplication rates of WT and Δ*clpB* in the whole blood of tilapia after 90 and 180 min. **D** Survival of WT and Δ*clpB* cocultured with tilapia HKLs for 2 and 3 h. **E** Colonization of the WT and Δ*clpB* strains in various tissues from tilapia. ns, not significant; *P < 0.05, **P < 0.01, ***P < 0.001.

To investigate whether ClpB affects GBS interactions with the host, we performed a range of adherence, invasion, and phagocytosis assays. We found that the deletion of *clpB* failed to reduce invasion into the TiB cells but resulted in a 63.7% reduction in adherence to the TiB cells (*P* < 0.05) (Figure 4A). The survival rates of the two strains in RAW264.7 cells were also investigated; these analyses showed that the viable counts of the Δ*clpB* strains were lower than those of the WT strain. At 4 h postengulfment, only approximately 54.3% of the ∆clpB strain survived at an MOI of 10; in contrast, the survival rate of the WT strain was 78.1% (*P* < 0.0001) (Figure 4B). These findings support the idea that ClpB is essential for survival within macrophages.

When whole blood and HKLs from tilapia were exposed to ΔclpB, we observed greater susceptibility to bactericidal impact and poorer viability ex vivo. In the whole-blood bactericidal assay, CFU counts for the two strains increased after 90 and 180 min of incubation, respectively, indicating that GBS could proliferate in the whole blood of tilapia. However, the multiplication rates were significantly lower in the *clpB* deletion strain (8.6-fold) than in the WT strain (14.4-fold; *P* < 0.001) (Figure 4C). When incubated with HKLs isolated from tilapia, the Δ*clpB* strain was more sensitive to leukocytes and showed a lower survival rate (14.0%) than the WT strain (45.8%; *P* < 0.001) (Figure 4D).

To gain a better understanding of the functionality of *clpB* during systemic infection, we evaluated the bacterial load in several organs (Figure 4E). At 12 h postinfection, the CFU of the Δ*clpB* strain was significantly lower than that of the WT strain, with a 3.7-fold reduction in the blood, a 1.5-fold reduction in the spleen, and a 2.5-fold reduction in the brain. These findings provide significant evidence that ClpB plays a crucial role in the pathogenesis of GBS.

**Figure 5:**
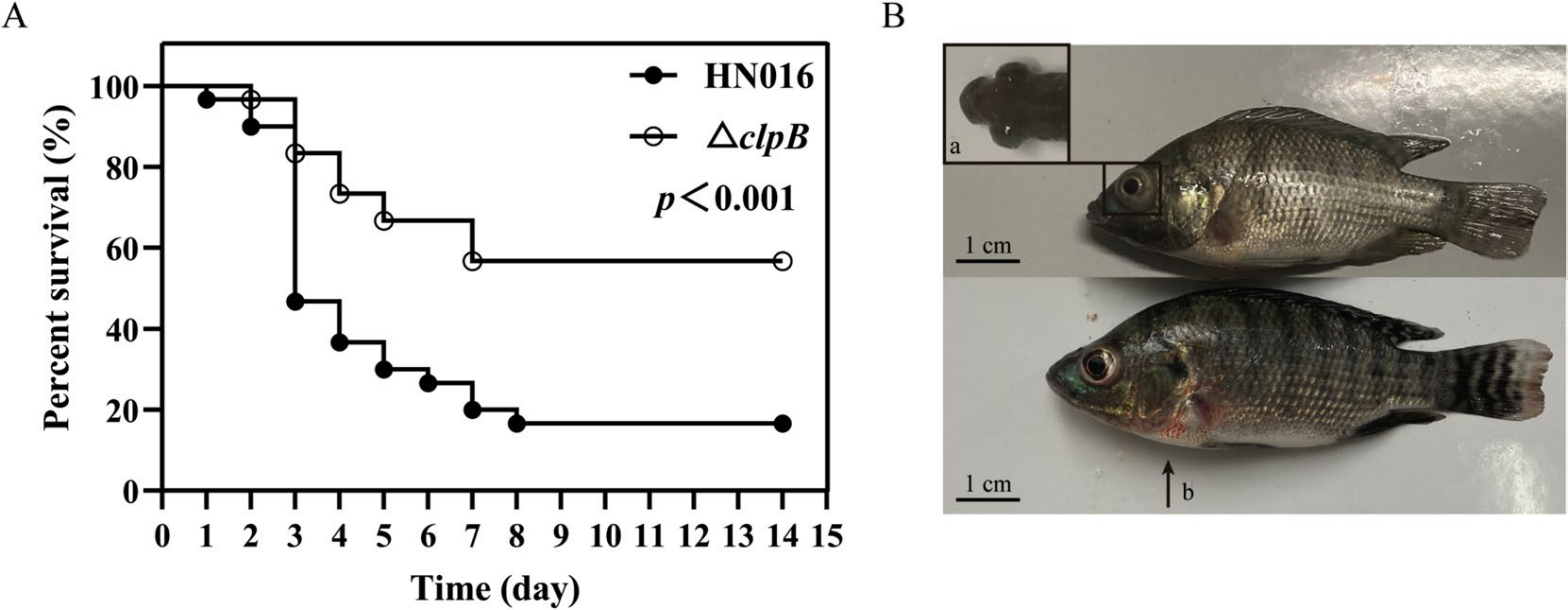
**Tilapia infection assay** **A** Survival curves of tilapia infected with the two strains (*n* = 30). **B** Clinical symptoms of tilapia. **a** exophthalmos; **b** skin lesion.

To investigate the effect of *clpB* inactivation on GBS virulence, we injected tilapia via the i.p*.* route with either WT or Δ*clpB* at a dose of 2 × 10^8^ CFU/fish (Figure 5A). Tilapia infected with WT rapidly succumbed, exhibiting exophthalmia, corneal opacity, and disorientation (Figure 5B), with a death rate of 50% within 72 h. In contrast, only 18.9% mortality occurred within 72 h in the Δ*clpB* infection group. At 14 d postinfection, 56.7% of the tilapia survived the Δ*clpB* challenge, whereas only 16.7% of tilapia in the WT infection group survived, indicating that Δ*clpB* has attenuated virulence in tilapia (Additional file 7).

### *clpB* gene deficiency caused changes in the bacterial transcriptome

**Figure 6:**
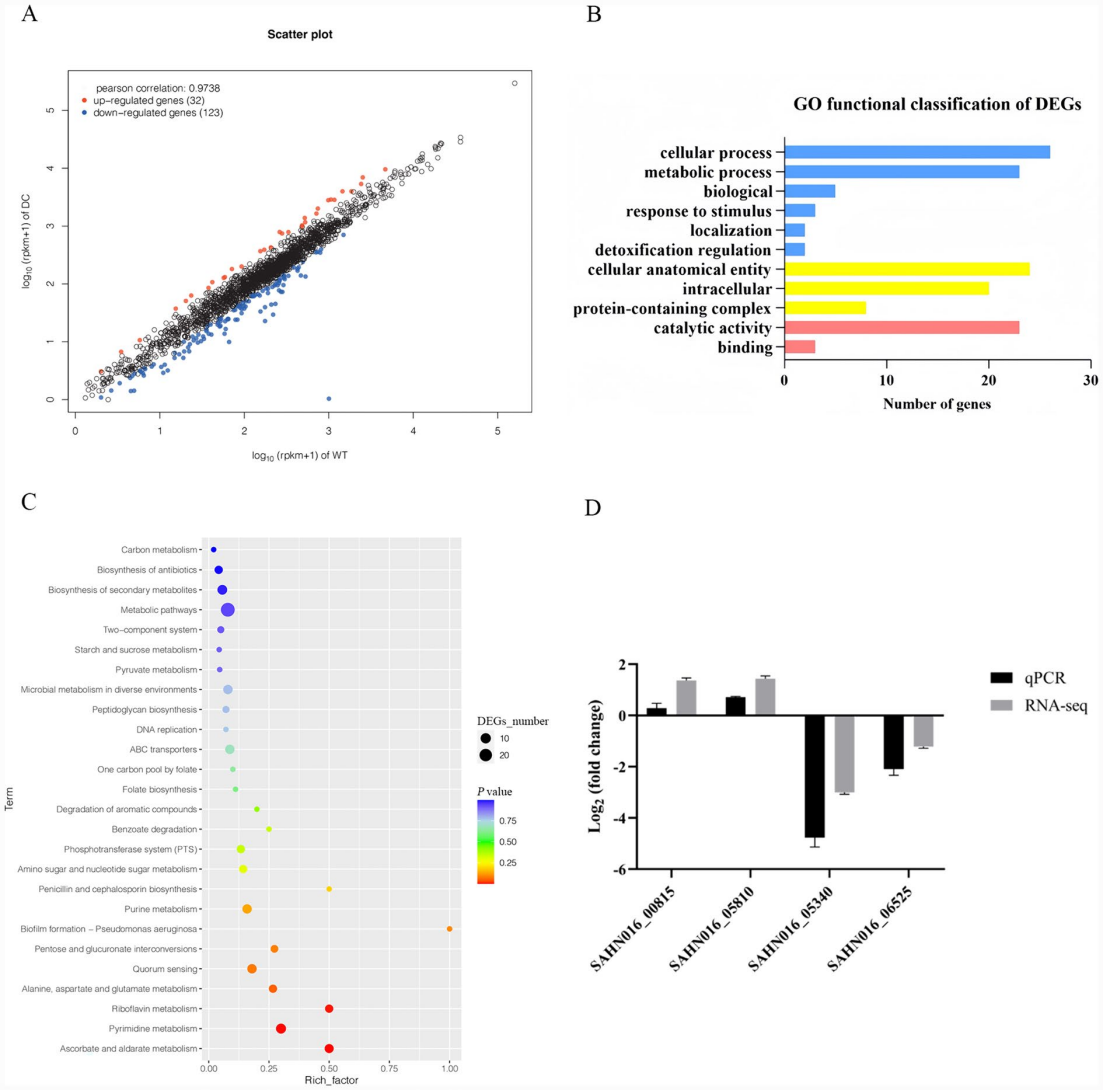
**Transcriptome analysis of GBS strains.** **A** Scatter plot showing DEGs. **B** GO functional classification of DEGs. (**C**) KEGG pathway analysis. **D** Verification of the transcriptome sequence.

Since our previous results revealed that *clpB* deletion significantly affected virulence, we next analysed the transcriptome of the *clpB* mutant to determine global changes in the whole genome. We compared the transcriptional profiles of the *clpB* mutant and WT strains after culture with RAW 264.7 cells. A total of 155 differentially expressed genes (DEGs) were identified in the Δ*clpB* strain (Additional file 8), with 32 upregulated genes and 123 downregulated genes (Figure 6A). The upregulated genes included several important stress response genes and genes that are known to be associated with intracellular survival in macrophages. These genes included the stress response regulator Gls24 (SAHN016_05815), the universal stress protein UspA (SAHN016_08150), and carbamoyl-phosphate synthase (SAHN016_05340), which have been confirmed to be involved in the response to environmental stress and intracellular survival. The downregulated genes were associated with multiple metabolic pathways.

Applying GO functional annotation, 149 DEGs were annotated to three major functions: biological processes (64, 43.0%), cellular components (52, 34.9%), and molecular functions (33, 22.1%) (Figure 6B). Kyoto Encyclopedia of Genes and Genomes (KEGG) enrichment analysis revealed that the top three categories of DEGs were related to ascorbate and aldarate metabolism, pyrimidine metabolism, and riboflavin metabolism pathways (Figure 6C).

Furthermore, we found that ClpB exerted regulatory effects on four genes known to be associated with intracellular survival in macrophages. The products of these four genes are pyridine nucleotide-disulfide oxidoreductase (SAHN016_00815), CsbD family protein (SAHN016_05810), carbamoyl-phosphate synthase (SAHN016_05340), and acetyltransferase (SAHN016_06525).

qRT‒PCR was used to validate the RNA‒seq results, and the agreement between the two sets of data confirmed that the results of the RNA‒seq analysis were reliable (Figure 6D). These findings suggest that *clpB* has regulatory effects on multiple metabolic pathways and that GBS compensates for the absence of *clpB* by promoting the expression of other stress response genes.

## Discussion

During infection, pathogens are inevitably exposed to numerous stressors, thus leading to the aggregation and accumulation of specific proteins [36, 37]. To overcome this challenge, pathogens have developed sophisticated molecular systems in which bacterial chaperone systems play a crucial role. These chaperones are essential for bacterial survival, especially under stressful conditions, such as in the presence of the host’s immune defence response, including inflammation [19]. ClpB, first identified as a heat shock protein in *E. coli*, is a protein disaggregate known to be important for survival during severe stress in various pathogens [38]. However, prior to this study, the specific functionality of ClpB in GBS patients remained largely unknown.

Previous analysis of the GBS HN016 genome sequence [39] identified the gene encoding the ClpB protease. Sequence alignment revealed that ClpB_GBS_ contains two conserved NBDs, the signature of class I AAA + proteases, whereas class II AAA + proteases such as ClpX have only one NBD [40]. To determine the distribution of ClpB, we undertook a systematic search for its homologues. ClpB_GBS_ shares identity not only with gram-positive bacteria (*Streptococcus pneumoniae*, *E. faecalis*, *Staphylococcus aureus*, and *Listeria monocytogenes)* but also with gram-negative bacteria (*E. coli* and *F. tularensis*), as well as *Mycobacterium tuberculosis*. Further investigation revealed that this homologue was extensively encoded in most GBS strains, indicating that it may function as a conserved protein chaperone to promote the ability of GBS to cope with stress and/or increase virulence.

To investigate the contribution of ClpB to GBS, we constructed a mutant strain (Δ*clpB*) in GBS HN016 harbouring a clpB deletion. The Δ*clpB* mutant showed an attenuated capacity to tolerate heat and low pH but not oxidative or osmotic stress, indicating that GBS ClpB is essential for acid resistance and thermotolerance; this was consistent with a previous study in *F. tularensis* subsp. *tularensis* SCHU S4 [20]. The *F. tularensis* subsp. *tularensis* SCHU S4 ΔclpB mutant exhibited extreme sensitivity to high temperature and low pH but not to oxidative stress (osmotic stress was not tested in this previous study). However, in the mediation of stress tolerance in various bacteria, ClpB proteins play a variety of roles. The deletion of *clpB* in *F. novicida* resulted in an extreme defect in survival only under heat pressure [20], while the deletion of clpB in *F. tularensis* ssp. *holarctica* rendered the strain highly sensitive to heat, ethanol, oxidation, and low pH [41]. Furthermore, *clpB* null mutants of *Salmonella enterica* serovar Typhimurium and *Vibrio cholerae* both exhibited increased susceptibility to heat and oxidative stress [42, 43]. In *M. tuberculosis*, the genetic depletion of *clpB* impaired bacterial recovery after exposure to host-like stresses, particularly reactive nitrogen species [18, 44]. Collectively, these results demonstrate that ClpB_GBS_ helps to mediate tolerance to some adverse conditions, particularly heat, but does not confer tolerance to all types of stress stimuli.

Streptococcal outbreaks in farmed tilapia tend to occur in hot summers when water temperatures rise [45, 46]. It has been reported that a water temperature above 26 ℃ can lead to the development of streptococcal outbreaks caused by GBS infection in tilapia under both natural [7, 46] and experimental conditions [47]. According to our stress tolerance assays, Δ*clpB* showed a significantly attenuated ability to tolerate heat, indicating the dominant contribution of ClpB to thermotolerance. Therefore, we hypothesized that during the infection process, GBS encounters heat conditions and is able to overcome heat shock via the heat shock protein ClpB. Furthermore, a high temperature that exceeds the optimum range for fish can also negatively impact their physiology, including that of the immune system [48, 49]. This hypothesis was supported by the fact that Δ*clpB* showed significantly attenuated mortality compared to that of WT tilapia when the water temperature was maintained at 34 ℃.

In addition to promoting stress tolerance, multiple important bacterial pathogens are known to utilize ClpB during infection. With this in mind, we performed a phagocytosis assay and a tilapia infection assay to compare the systemic infection capacity of Δ*clpB* with that of the parent strain. We found that the Δ*clpB* mutant showed impaired intracellular viability in macrophages. The acidic lysosomes in macrophages are engaged in the digestion and clearance of invading streptococci, and the low pH within these lysosomes results in the elimination of engulfed bacteria [50]. In our stress tolerance assays, Δ*clpB* also exhibited a significant reduction in response to acidic stress. GBS lacking *clpB* exhibited increased sensitivity to acidic pH, which subsequently resulted in reduced intracellular viability in macrophages. Similarly, a previous study showed that the CovS/R system, a crucial two-component system, improved the intracellular survival and resilience of macrophages to pH stress caused by GBS [14]. GBS successfully evades multiple antimicrobial agents in the blood via its intracellular location in macrophages; this process is crucial for the development of bacteremia and subsequent meningitis [51]. Indeed, the bacterial load in the brains of tilapia infected with Δ*clpB* was significantly reduced, indicating that Δ*clpB* has a weakened ability to breach the blood‒brain barrier (BBB). According to the “Trojan horse” theory, *Streptococcus suis* is engulfed by macrophages, allowing the bacteria to persist and circulate intracellularly [52, 53]. Moreover, *Piscirickettsia salmonis* was demonstrated to significantly upregulate ClpB expression during intramacrophage growth, thus demonstrating that ClpB allows bacteria to conform to adverse intracellular environments and promotes replication [54].

In our tilapia infection assay, Δ*clpB* showed poor colonization in the blood, indicating that Δ*clpB* is more easily eliminated by antimicrobial agents in the blood. As a result, clpB deficiency had a negative impact on the ability of GBS to spread from the bloodstream to other tissues, such as the brain, liver, and kidney. A previous study involving tilapia reported that GBS infection caused differing degrees of degeneration and necrosis in the liver, spleen, kidney, eyes, and brain [55]. These earlier findings were consistent with those from our tilapia infection assay, where the burden created by the Δ*clpB* mutation in the spleens and brains of tilapia was significantly lower than that in the WT. These findings demonstrated the crucial role of ClpB in bacterial tolerance to acidic pH, contributing to GBS survival in vivo.

These findings raised the question of whether the crucial and ostensibly conserved role of ClpB in GBS virulence results from its involvement in managing stress or from a more direct role in controlling virulence factors. We compared the transcriptional profiles of the strains to clarify the impacts of ClpB on the transcriptome. Our analyses indicated that ClpB exerts regulatory effects on key metabolic pathways, including the ascorbate and aldarate, pyrimidine, and riboflavin metabolism pathways. Similarly, in the case of *P. gingivalis*, 136 genes were differentially regulated in the *clpB* mutant according to the overall genetic profile. Based on the regulatory pattern of genes, functional classifications were created, including the cell envelope and the biosynthesis of proteins, purines, pyrimidines, nucleosides, and nucleotides [56]. Furthermore, in *Leptospira interrogans*, the majority of ClpB-interacting proteins were shown to be involved in key metabolic pathways, including glycolysis-gluconeogenesis, the tricarboxylic acid (TCA) cycle, and amino acid and fatty acid metabolism [19]. Metabolic processes, especially nucleotide biosynthesis, have an important influence on bacterial growth and virulence. It is necessary for the majority of free-living organisms to produce and/or acquire purines and pyrimidines, the building blocks of nucleotides, to exist [57]. Bacterial growth in human blood requires the biosynthesis of purines and pyrimidines, as demonstrated in *E. coli*, *S. typhimurium*, and *Bacillus anthracis* [58]. In addition to its role in metabolism, our transcriptomic analysis revealed that ClpB regulates four genes known to be associated with intracellular survival in macrophages, whose products are pyridine nucleotide-disulfide oxidoreductase, the CsbD family protein, carbamoyl-phosphate synthase, and acetyltransferase. Pyridine nucleotide-disulfide oxidoreductase, also known as SAHN016_00815, is involved in disulfide oxidoreductase activity and electron transport [51]. According to a previous transcriptomic analysis, the expression of pyridine nucleotide-disulfide oxidoreductase was downregulated in GBS patients during vaginal colonization [59]. However, in our present investigation, the expression of this gene was upregulated in the Δ*clpB* strain compared to the WT strain, suggesting that the deletion of *clpB* prevents GBS from downregulating *clpB* in response to stressful conditions. The CsbD family protein, also known as SAHN016_05810, is a bacterial protein produced in response to general stress [60]; however, there are no reports related to the function of CsbD-like proteins in GBS. In *Bacillus subtilis*, the CsbD family of proteins manipulates the expression of general stress genes [61]. Thus, we assume that SAHN016_05810 is elevated to compensate for the absence of ClpB. Using a hypersensitive *E. coli* genetic system, Park et al. [62] demonstrated that carbamoyl-phosphate synthase was implicated in resistance to nitrosative stress caused by reactive nitrogen intermediates. In addition, SAHN016_05340, also known as carbamoyl-phosphate synthase, was downregulated, thus resulting in poor intracellular survival of Δ*clpB* in macrophages.

Lysine acetylation is a key metabolic regulatory process in prokaryotes and is carried out enzymatically by the protein acetyltransferase, which transfers acetyl groups in a specific manner. Acetyltransferase, identified as ActG in *Streptococcus mutans*, was previously shown to interfere with water-insoluble EPS synthesis and biofilm formation in *S. mutans* [63, 64]. The downregulation of SAHN016_06525, encoding acetyltransferase, may be responsible for the metabolic changes observed in Δ*clpB*.

Furthermore, our qRT‒PCR results showed trends similar to those of the RNA‒seq results, indicating that the results of the RNA‒seq analysis were reliable. According to these findings, we believe that ClpB contributes to the virulence of GBS by promoting energy homeostasis and regulating the expression of several genes associated with the stress response.

Overall, our study identified a molecular chaperone, ClpB, that is involved in stress tolerance and virulence in *S. agalactiae*. We generated a Δ*clpB* mutant that exhibited a defective capacity to confer tolerance to certain stress stimuli in vitro, including heat and low pH. Moreover, we proved that Δ*clpB* exhibited defective intracellular replication ex vivo and weakened systemic infection in vivo. Furthermore, the deletion of *clpB* changed the expression levels of numerous genes and subsequently impacted several cellular processes. These results provide new perspectives on the functionality of molecular chaperones in the virulence of *S. agalactiae.* Considering its high levels of conservation in bacteria and its absence in animals, ClpB also has potential as a target for developing new antimicrobial treatments for treating bacterial infections.

### Supplementary Information

**Additional file 1 Ramachandran plot of ClpB Swiss-Models.** Red regions indicate the most favoured region, yellow regions indicate the additional allowed region, and light yellow regions indicate the generously allowed region. The black dots indicate the individual amino acids that make up the protein.

**Additional file 2 Fold change in mRNA expression.** Relative mRNA expression levels of the clpB upstream, downstream, and clpB genes in the WT and ΔclpB strains. The value of the target genes in the WT was set to 1.0. “ns” and “***” indicate “*P* > 0.05” and “*P* < 0.001”, respectively. 23.

**Additional file 3 Bacterial strains, plasmids, and cell lines.** Summary of the bacterial strains, plasmids, and cell lines used in this study.

**Additional file 4 Databases and websites used in this study.** Database and bioinformatics analysis software and websites.

**Additional file 5 Primers.** Primers used in this study.

**Additional file 6 ClpB identity in various GBS strains.** The presence of ClpB in various GBS strains.

**Additional file 7 Cumulative number of deaths of tilapia infected with GBS strains.** Death of tilapia infected with GBS strains.

**Additional file 8 Differentially expressed genes (DEGs) in the ΔclpB strain.**

## Data Availability

The datasets used and/or analysed during the current study are available from the corresponding author upon reasonable request.
