# Supplementary figures and images for: Identification of ClpB, a molecular chaperone involved in the stress tolerance and virulence of Streptococcus agalactiae
Source: Vet Res. 2024 May 15;55:60. doi: 10.1186/s13567-024-01318-6 (PMC11094935; doi:10.1186/s13567-024-01318-6)

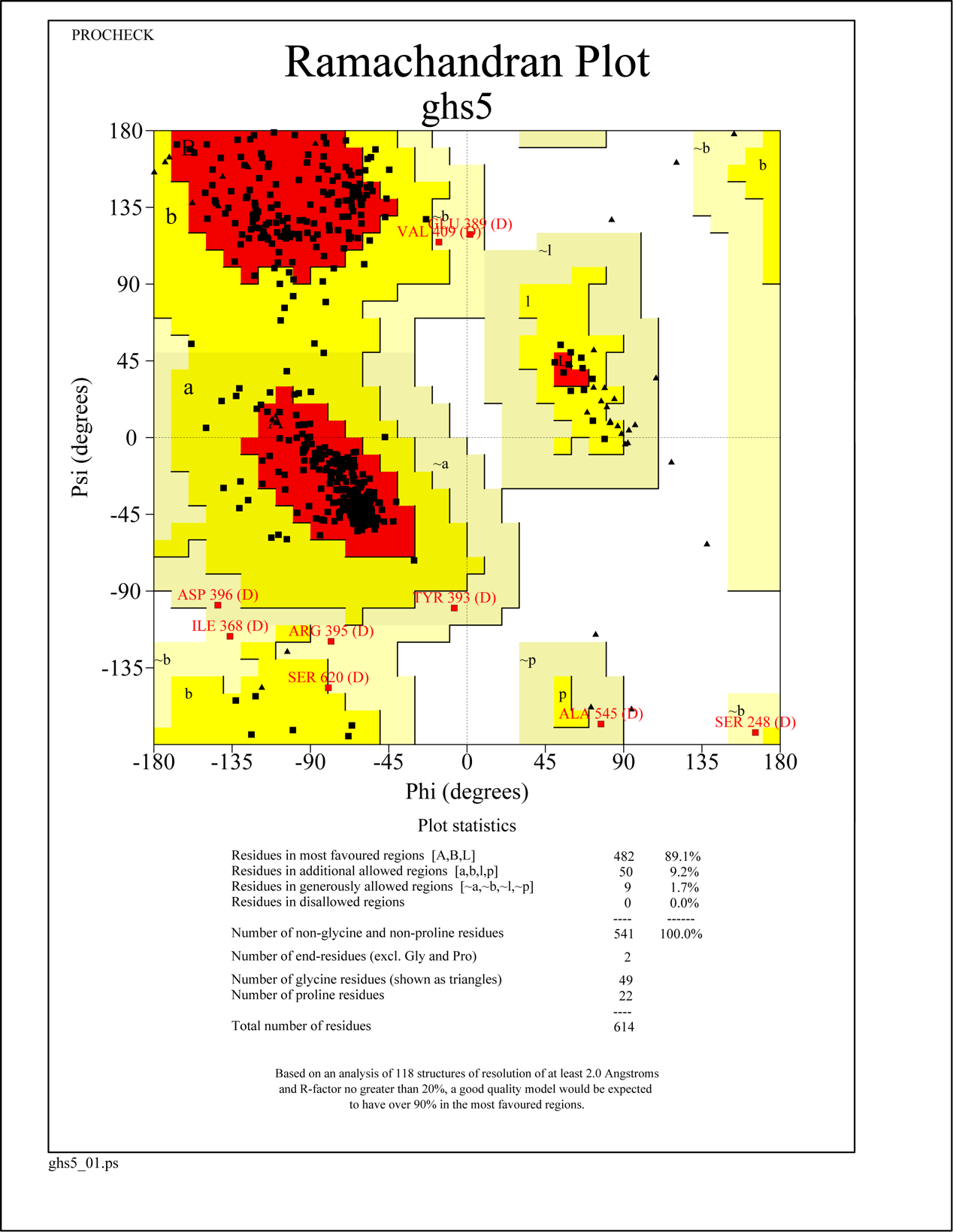

Supplement: Supplementary file 1 — Additional file 1 Ramachandran plot of ClpB Swiss-Models. Red regions indicate the most favoured region, yellow regions indicate the additional allowed region, and light yellow regions indicate the generously allowed region. The black dots indicate the individual amino acids that make up the protein. [file 13567_2024_1318_MOESM1_ESM.docx]

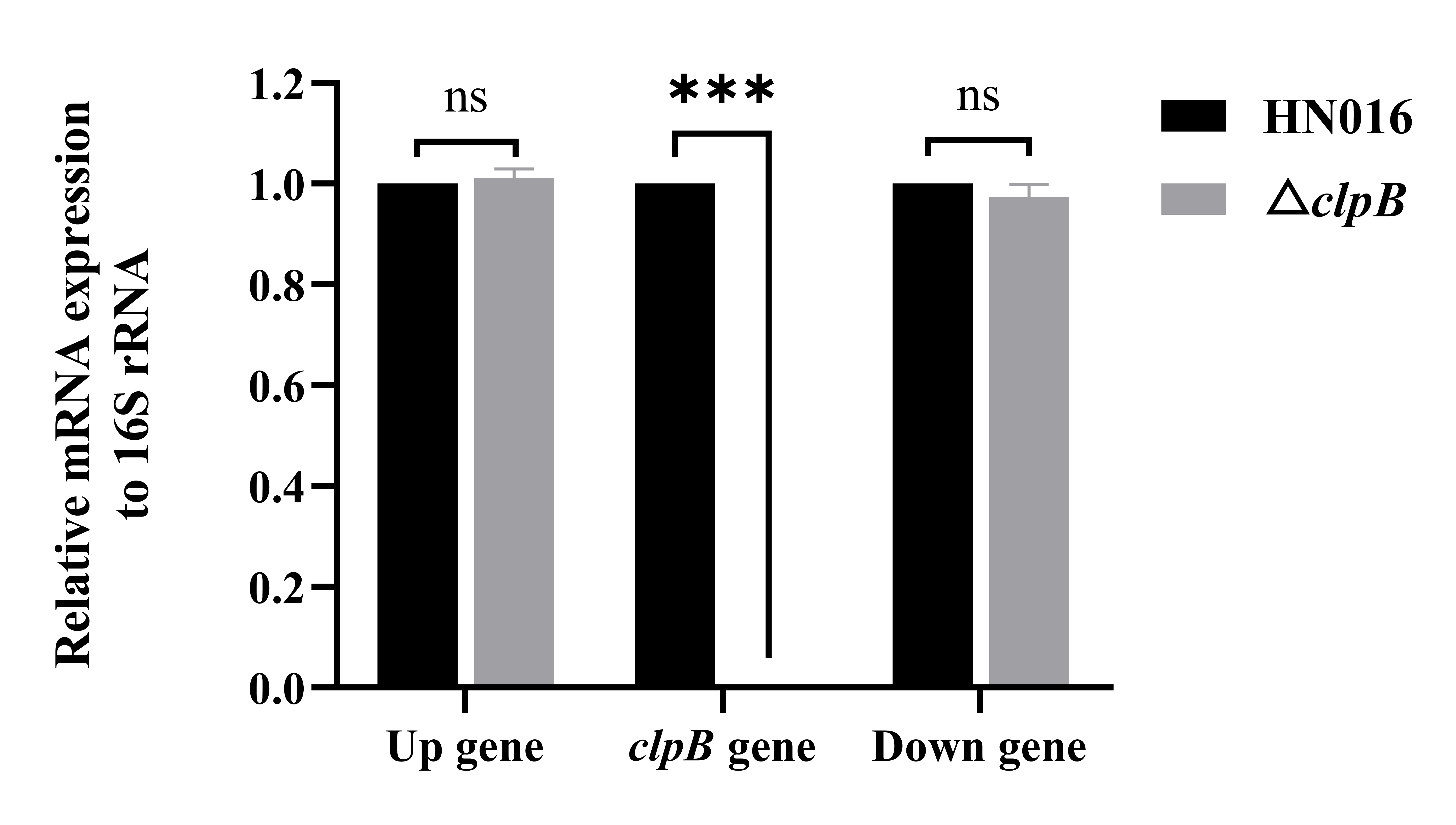

Supplement: Supplementary file 2 — Additional file 2 Fold change in mRNA expression. Relative mRNA expression levels of the clpB upstream, downstream, and clpB genes in the WT and ΔclpB strains. The value of the target genes in the WT was set to 1.0. “ns” and “***” indicate “P > 0.05” and “P < 0.001”, respectively. 23. [file 13567_2024_1318_MOESM2_ESM.docx]
