# Supplementary material for: Identification of ClpB, a molecular chaperone involved in the stress tolerance and virulence of Streptococcus agalactiae
Source: Vet Res. 2024 May 15;55:60. doi: 10.1186/s13567-024-01318-6 (PMC11094935; doi:10.1186/s13567-024-01318-6)
Supplement: Supplementary file 3 — Additional file 3 Bacterial strains, plasmids, and cell lines. Summary of the bacterial strains, plasmids, and cell lines used in this study. [file 13567_2024_1318_MOESM3_ESM.docx]

**Additional file 3.** **Bacterial strains, plasmids, and cell lines.** Summary of the bacterial strains, plasmids, and cell lines used in this study.

| **Strain or plasmid or cells** | **Description** | **Source or reference** |
| --- | --- | --- |
| Strains |  |  |
| *S. agalactiae* |  |  |
| HN016 | A virulent strain of *S. agalactiae* isolated from tilapia with meningoencephalitis in China | [61] |
| Δ*clpB* mutant | *clpB* gene deletion mutant of HN016 | This study |
| *E. coil* DH5α | Used for production of recombinant plasmids | Trans |
| Plasmids |  |  |
| pSET4s | Thermosensitive suicide vector for gene replacement in Streptococcus replication; Spc^r^ | [57] |
| pSET4s-*clpB* | Recombinant vector with pSET4s background, designed for knockout of *clpB*; Spc^r^ | This study |
| Cells |  |  |
| RAW264.7 | Mouse leukemia cells of monocyte macrophage | [26] |
| TiB | Tilapia brain cells line | [62] |

Spc^r^, Spectinomycin resistant.
