# Supplementary material for: Identification of ClpB, a molecular chaperone involved in the stress tolerance and virulence of Streptococcus agalactiae
Source: Vet Res. 2024 May 15;55:60. doi: 10.1186/s13567-024-01318-6 (PMC11094935; doi:10.1186/s13567-024-01318-6)
Supplement: Supplementary file 4 — Additional file 4 Databases and websites used in this study. Database and bioinformatics analysis software and websites. [file 13567_2024_1318_MOESM4_ESM.docx]

**Additional file 4 Databases and Websites used in this study.** Database and bioinformatics analysis software and websites.

| **Software** | **Websites** |
| --- | --- |
| NCBI | https://www. ncbi.nlm.nih.gov/ |
| Clustal Omega software online | https://www.ebi.ac.uk/Tools/msa/clustalo/ |
| ESPript 3.0 | https://espript. ibcp.fr/ESPript/ESPript/index.php |
| Swiss-Model | https://swissmodel.expasy.org/interactive |
| PROCHECK | http://www.csb.yale.edu/userguides/datamanip/procheck/manual/index.html |
