# Supplementary material for: Identification of ClpB, a molecular chaperone involved in the stress tolerance and virulence of Streptococcus agalactiae
Source: Vet Res. 2024 May 15;55:60. doi: 10.1186/s13567-024-01318-6 (PMC11094935; doi:10.1186/s13567-024-01318-6)
Supplement: Supplementary file 5 — Additional file 5 Primers. Primers used in this study. [file 13567_2024_1318_MOESM5_ESM.docx]

**Additional file 5** **Primers.** Primers used in this study.

| **Primers** | **Sequence (5'-3')** | **Function** |
| --- | --- | --- |
| *clpB*-A | CGCGTCGACCAATTGTTGCGGAATAACCT | Left fragment of *clpB* |
| *clpB*-B | AGATAGTAATCTGTGATAGC TGAGAATCCAAGCGAAAAAG |  |
| *clpB*-C | GCTATCACAGATTACTATCT GAGCATAGGCTACCCCTTTC | Right fragment of *clpB* |
| *clpB*-D | TCC CCCGGG GCGATAAGGTCAATGCTGTC |  |
| *clpB*-E | GGTGTATCTTGATCGTCGACC | A inner fragment of *clpB*; used to confirm the deletion of *clpB* by PCR |
| *clpB*-F | GCGAAGGTAGTATGGATGCAG |  |
| *clpB*-G | CGCGTCGACCAATTGTTGCGGAATAACCT | An external fragment of *clpB*; used to confirm the deletion of *clpB* by PCR |
| *clpB*-H | TCC CCCGGG GCGATAAGGTCAATGCTGTC |  |
| *clpB*-up-F | TGGCGACTGATGGTGGTAA | The upstream gene of *clpB* for qRT-PCR |
| *clpB*-up-R | TTGCGATAAGGTCAATGCTGT |  |
| *clpB*-self-F | TCTGAGTCACGTCCAATAACAG | *clpB* gene for qRT-PCR |
| *clpB*-self-R | CAACTCCTTCTCAAGCCAAAG |  |
| *clpB*-down-F | GGCGACTGATGGTGGTAAT | The downstream gene of *clpB* for qRT-PCR |
| *clpB*-down-R | TTGCGATAAGGTCAATGCTGT |  |
