# Supplementary material for: Identification of ClpB, a molecular chaperone involved in the stress tolerance and virulence of Streptococcus agalactiae
Source: Vet Res. 2024 May 15;55:60. doi: 10.1186/s13567-024-01318-6 (PMC11094935; doi:10.1186/s13567-024-01318-6)
Supplement: Supplementary file 6 — Additional file 6 ClpB identity in various GBS strains. The presence of ClpB in various GBS strains. [file 13567_2024_1318_MOESM6_ESM.docx]

**Additional file 6** **ClpB identity in various GBS strains.** The presence of ClpB in various GBS strains.

| **Bacterial species** | **Accession** | **Gene sequence identity (%)^a^** |
| --- | --- | --- |
| *Streptococcus agalactiae* | MCK6353350.1 | 100 |
| *Streptococcus agalactiae* | MCC9699698.1 | 100 |
| *Streptococcus agalactiae* | MCC9725189.1 | 100 |
| *Streptococcus agalactiae* | MCC9818205.1 | 100 |
| *Streptococcus* | WP_000882549.1 | 100 |
| *Streptococcus agalactiae* | WP_060458513.1 | 99.87 |
| *Streptococcus agalactiae* | WP_000882553.1 | 99.87 |
| *Streptococcus agalactiae* | WP_065736151.1 | 99.87 |
| *Streptococcus agalactiae* | WP_141444603.1 | 99.87 |
| *Streptococcus agalactiae* | WP_047199713.1 | 99.87 |
| *Streptococcus agalactiae* | WP_070009720.1 | 99.87 |
| *Streptococcus agalactiae* | WP_137191985.1 | 99.87 |
| *Streptococcus agalactiae* | WP_017649473.1 | 99.87 |
| *Streptococcus agalactiae* | WP_065733426.1 | 99.87 |
| *Streptococcus agalactiae* | WP_000882550.1 | 99.87 |
| *Streptococcus agalactiae* | MCC9827433.1 | 99.87 |
| *Streptococcus agalactiae* | WP_069988820.1 | 99.87 |
| *Streptococcus agalactiae* | WP_256644796.1 | 99.87 |
| *Streptococcus agalactiae* | WP_050149996.1 | 99.87 |
| *Streptococcus agalactiae* | WP_000882547.1 | 99.87 |
| *Streptococcus agalactiae* | MCC9961908.1 | 99.87 |
| *Streptococcus agalactiae* | WP_161551895.1 | 99.87 |
| *Streptococcus agalactiae* | WP_047199731.1 | 99.87 |
| *Streptococcus agalactiae* | WP_237395043.1 | 99.87 |
| *Streptococcus agalactiae* | MCC9952711.1 | 99.87 |
| *Streptococcus agalactiae* | MCC9943690.1 | 99.87 |
| *Streptococcus agalactiae* | MCC9963533.1 | 99.87 |
| *Streptococcus agalactiae* | WP_228380212.1 | 99.86 |
| *Streptococcus agalactiae* | MCC9869959.1 | 99.85 |
| *Streptococcus agalactiae* | WP_248129258.1 | 99.73 |
| *Streptococcus agalactiae* | WP_165736610.1 | 99.73 |
| *Streptococcus agalactiae* | MCC9941726.1 | 99.73 |
| *Streptococcus agalactiae* | WP_161516605.1 | 99.73 |
| *Streptococcus* | WP_000882546.1 | 99.73 |
| *Streptococcus agalactiae* | MCC9900183.1 | 99.73 |
| *Streptococcus agalactiae* | MCC9694780.1 | 99.73 |
| *Streptococcus agalactiae* | MCC9692640.1 | 99.73 |
| *Streptococcus agalactiae* | MCC9904839.1 | 99.73 |
| *Streptococcus agalactiae* 515 | EAO70429.1 | 99.71 |
| *Streptococcus* | WP_000882544.1 | 99.6 |
| *Streptococcus agalactiae* | WP_016502611.1 | 99.6 |
| *Streptococcus agalactiae* | WP_017766981.1 | 99.6 |
| *Streptococcus agalactiae* | MCC9703093.1 | 99.6 |
| *Streptococcus agalactiae* | WP_165695757.1 | 99.6 |
| *Streptococcus agalactiae* | WP_017645303.1 | 99.6 |
| *Streptococcus agalactiae* | WP_060457657.1 | 99.6 |
| *Streptococcus agalactiae* | WP_270988270.1 | 99.6 |
| *Streptococcus agalactiae* | WP_017650651.1 | 99.6 |
| *Streptococcus agalactiae* | WP_192802550.1 | 99.6 |
| *Streptococcus agalactiae* | WP_055318207.1 | 99.6 |
| *Streptococcus agalactiae* | WP_192801178.1 | 99.6 |
| *Streptococcus agalactiae* | WP_134224358.1 | 99.6 |
| *Streptococcus agalactiae* | MCC9814345.1 | 99.6 |
| *Streptococcus agalactiae* | MCK6377235.1 | 99.6 |
| *Streptococcus agalactiae* | MCC9874206.1 | 99.59 |
| *Streptococcus agalactiae* | MCC9717651.1 | 99.59 |
| *Streptococcus agalactiae* | MCC9880777.1 | 99.57 |
| *Streptococcus agalactiae* | MCC9743149.1 | 99.57 |
| *Streptococcus agalactiae* | WP_270981287.1 | 99.47 |
| *Streptococcus agalactiae* | WP_264402208.1 | 99.47 |
| *Streptococcus agalactiae* | WP_070043539.1 | 99.47 |
| *Streptococcus agalactiae* | WP_017647074.1 | 99.47 |
| *Streptococcus agalactiae* | WP_016480424.1 | 99.47 |
| *Streptococcus agalactiae* | WP_017643903.1 | 99.47 |
| *Streptococcus agalactiae* | WP_070001244.1 | 99.47 |
| *Streptococcus agalactiae* | WP_000882545.1 | 99.47 |
| *Streptococcus agalactiae* | WP_069989424.1 | 99.47 |
| *Streptococcus agalactiae* | WP_000882543.1 | 99.47 |
| *Streptococcus agalactiae* | WP_088197829.1 | 99.47 |
| *Streptococcus agalactiae* | WP_000882542.1 | 99.47 |
| *Streptococcus agalactiae* | WP_069989823.1 | 99.47 |
| *Streptococcus* | WP_000882548.1 | 99.47 |
| *Streptococcus agalactiae* | WP_017643525.1 | 99.47 |
| *Streptococcus agalactiae* | WP_017644450.1 | 99.47 |
| *Streptococcus agalactiae* | WP_017771736.1 | 99.47 |
| *Streptococcus agalactiae* | AUO83281.1 | 99.47 |
| *Streptococcus agalactiae* | MCC9980959.1 | 99.47 |
| *Streptococcus agalactiae* | AFV71949.1 | 99.47 |
| *Streptococcus agalactiae* | MCA5916074.1 | 99.46 |
| *Streptococcus agalactiae* | MCK6368811.1 | 99.46 |
| *Streptococcus agalactiae* | MCC9872337.1 | 99.43 |
| *Streptococcus agalactiae* | MCC9735174.1 | 99.41 |
| *Streptococcus agalactiae* 138P | AHN30102.1 | 99.34 |
| *Streptococcus agalactiae* | MCC9914293.1 | 99.34 |
| *Streptococcus agalactiae* | WP_060800991.1 | 99.34 |
| *Streptococcus agalactiae* | WP_017648399.1 | 99.34 |
| *Streptococcus agalactiae* | WP_017646718.1 | 99.34 |
| *Streptococcus agalactiae* | MCD0123227.1 | 99.34 |
| *Streptococcus agalactiae* | WP_230227592.1 | 99.34 |
| *Streptococcus agalactiae* | WP_017767395.1 | 99.34 |
| *Streptococcus agalactiae* | WP_050198387.1 | 99.34 |
| *Streptococcus agalactiae* | AUO98190.1 | 99.34 |
| *Streptococcus agalactiae* SS1219 | CCQ75876.1 | 99.34 |
| *Streptococcus agalactiae* | WP_161508491.1 | 99.34 |
| *Streptococcus agalactiae* | ODG95898.1 | 99.32 |
| *Streptococcus agalactiae* | MCD0152417.1 | 99.28 |
| *Streptococcus agalactiae* | MCC9877345.1 | 99.28 |
| *Streptococcus agalactiae* | WP_000882551.1 | 99.2 |
| *Streptococcus agalactiae* | WP_109867764.1 | 99.07 |
| *Streptococcus agalactiae* | WP_047212124.1 | 98.8 |

^a^ Gene sequence identity is compared with the ClpB sequence of GBS HN016 strain.
