# Supplementary material for: Identification of ClpB, a molecular chaperone involved in the stress tolerance and virulence of Streptococcus agalactiae
Source: Vet Res. 2024 May 15;55:60. doi: 10.1186/s13567-024-01318-6 (PMC11094935; doi:10.1186/s13567-024-01318-6)
Supplement: Supplementary file 7 — Additional file 7 Cumulative number of deaths of tilapia infected with GBS strains. Death of tilapia infected with GBS strains. [file 13567_2024_1318_MOESM7_ESM.docx]

**Additional file 7: Cumulative number of deaths of tilapia with GBS strains infection.** Death of tilapia by infected with GBS strains.

| **Group** | **1 d** | **2 d** | **3 d** | **4 d** | **5 d** | **6 d** | **7 d** | **8 d** | **9 d** | **10 d** | **11 d** | **12 d** | **13 d** | **14 d** | **Total death** | **Mortality rate** |
| --- | --- | --- | --- | --- | --- | --- | --- | --- | --- | --- | --- | --- | --- | --- | --- | --- |
| **HN016** | 1 | 2 | 13 | 3 | 2 | 1 | 2 | 1 | 0 | 0 | 0 | 0 | 0 | 0 | 25 | 83.30% |
| **∆*clpB*** | 0 | 1 | 4 | 3 | 2 | 0 | 3 | 0 | 0 | 0 | 0 | 0 | 0 | 0 | 13 | 43.30% |
